# Assessment of the Combined Effect of Epstein–Barr Virus and *Plasmodium falciparum* Infections on Endemic Burkitt Lymphoma Using a Multiplex Serological Approach

**DOI:** 10.3389/fimmu.2017.01284

**Published:** 2017-10-26

**Authors:** Ruth Aguilar, Delphine Casabonne, Cristina O’Callaghan-Gordo, Marta Vidal, Joseph J. Campo, Nora Mutalima, Evelina Angov, Sheetij Dutta, Deepak Gaur, Chetan E. Chitnis, Virander Chauhan, Angelika Michel, Silvia de Sanjosé, Tim Waterboer, Manolis Kogevinas, Rob Newton, Carlota Dobaño

**Affiliations:** ^1^ISGlobal, Barcelona Centre for International Health Research (CRESIB), Hospital Clínic, Universitat de Barcelona, Barcelona, Spain; ^2^CIBER Epidemiología y Salud Pública (CIBERESP), Madrid, Spain; ^3^Unit of Infections and Cancer, Cancer Epidemiology Research Programme, IDIBELL, Institut Català d’Oncologia, L’Hospitalet de Llobregat, Spain; ^4^ISGlobal, Center for Research in Environmental Epidemiology (CREAL), Barcelona, Spain; ^5^Universitat Pompeu Fabra (UPF), Barcelona, Spain; ^6^Department of Orthopaedic Surgery, Monash Health, Melbourne, VIC, Australia; ^7^Department of Surgery, School of Clinical Sciences, Monash University, Melbourne, VIC, Australia; ^8^WRAIR, Silver Spring, MD, United States; ^9^ICGEB, Delhi, India; ^10^School of Biotechnology, Jawaharlal Nehru University, New Delhi, India; ^11^German Cancer Research Center (DKFZ), Heidelberg, Germany; ^12^IMIM (Hospital del Mar Medical Research Institute), Barcelona, Spain; ^13^Epidemiology and Cancer Statistics Group, University of York, York, United Kingdom; ^14^MRC/UVRI Uganda Research Unit on AIDS, Entebbe, Uganda

**Keywords:** endemic Burkitt lymphoma, Epstein–Barr virus, *Plasmodium falciparum*, IgG, IgM, multiplex, children, Africa

## Abstract

Epstein–Barr virus (EBV) is a necessary cause of endemic Burkitt lymphoma (eBL), while the role of *Plasmodium falciparum* in eBL remains uncertain. This study aimed to generate new hypotheses on the interplay between both infections in the development of eBL by investigating the IgG and IgM profiles against several EBV and *P. falciparum* antigens. Serum samples collected in a childhood study in Malawi (2005–2006) from 442 HIV-seronegative children (271 eBL cases and 171 controls) between 1.4 and 15 years old were tested by quantitative suspension array technology against a newly developed multiplex panel combining 4 EBV antigens [Z Epstein–Barr replication activator protein (ZEBRA), early antigen-diffuse component (EA-D), EBV nuclear antigen 1, and viral capsid antigen p18 subunit (VCA-p18)] and 15 *P. falciparum* antigens selected for their immunogenicity, role in malaria pathogenesis, and presence in different parasite stages. Principal component analyses, multivariate logistic models, and elastic-net regressions were used. As expected, elevated levels of EBV IgG (especially against the lytic antigens ZEBRA, EA-D, and VCA-p18) were strongly associated with eBL [high vs low tertile odds ratio (OR) = 8.67, 95% confidence interval (CI) = 4.81–15.64]. Higher IgG responses to the merozoite surface protein 3 were observed in children with eBL compared with controls (OR = 1.29, 95% CI = 1.02–1.64), showing an additive interaction with EBV IgGs (OR = 10.6, 95% CI = 5.1–22.2, *P* = 0.05). Using elastic-net regression models, eBL serological profile was further characterized by lower IgM levels against *P. falciparum* preerythrocytic-stage antigen CelTOS and EBV lytic antigen VCA-p18 compared with controls. In a secondary analysis, abdominal Burkitt lymphoma had lower IgM to EBV and higher IgG to EA-D levels than cases with head involvement. Overall, this exploratory study confirmed the strong role of EBV in eBL and identified differential IgG and IgM patterns to erythrocytic vs preerythrocytic *P. falciparum* antigens that suggest a more persistent/chronic malaria exposure and a weaker IgM immune response in children with eBL compared with controls. Future studies should continue exploring how the malaria infection status and the immune response to *P. falciparum* interact with EBV infection in the development of eBL.

## Introduction

Endemic Burkitt lymphoma (eBL), a highly aggressive B-cell non-Hodgkin lymphoma, is one of the most prevalent pediatric cancers in areas of sub-Saharan Africa where *Plasmodium falciparum* transmission is high. Epstein–Barr virus (EBV) is found in almost all cases of eBL and is a necessary agent in the development of the tumor (1). eBL peaks at age 6–7 years, is more frequent in males, and the most common sites of presentation are jaws and abdomen. The occurrence of eBL is limited to *P. falciparum* holoendemic areas (2), and there is strong evidence that the parasite reactivates EBV both *in vivo* and *in vitro* (3, 4).

*P. falciparum*-specific antigens cause polyclonal B-cell activation and increase EBV-infected B-cell survival (5). The parasite deregulates the activation-induced deaminase enzyme that is associated with somatic hypermutation and class switch recombination of immunoglobulin genes. This leads to DNA damage and increases the likelihood of c-myc translocations that, when happening in EBV-infected cells, leads to the development of lymphoma (6). In line with these observations, the International Agency for Research on Cancer categorized *P. falciparum* infection as a probable carcinogen (group 2A) in relation to the pathogenesis of eBL (7).

The intensity and timing of EBV and *P. falciparum* infections seem important features in eBL etiology. *P. falciparum* has been associated with earlier EBV acquisition and virus reactivation (8) and increases the frequency and duration of EBV events (9). Past and present exposure to infections in relation to eBL can be evaluated using serological markers. Four prior case–control studies in Uganda, Malawi, Kenya and Ghana measured *P. falciparum* antibodies to evaluate associations between malaria infection history and eBL (10–13). Those studies assessed IgGs against (i) schizont extract by indirect immunofluorescence assay (10) or ELISA (11), (ii) histidin rich protein II, circumsporozoite surface protein (CSP-NANP_6_), SE36 subunit of serine repeat antigen-5 protein, and merozoite surface protein 1 (MSP-1_42_) by ELISA (13), and (iii) MSP-1, liver stage antigen 1 (LSA-1), and apical membrane antigen 1 (AMA-1) (12) by quantitative suspension array technology (qSAT). Serological analyses based on few antigens in Uganda and Malawi suggested that EBV and malaria act jointly on eBL, but their combined effect was not evaluated in Kenya and Ghana.

*P. falciparum* is a complex parasite with 5,000–6,000 predicted proteins and with a life cycle that comprises morphologically, sexual and antigenically distinct stages (preerythrocytic and erythrocytic) that are targeted by stage-specific immunity. Some antigenic epitopes of immunity are known, but there is not yet a validated set of proteins that can accurately indicate recent or past malaria exposure. A larger panel of antigens than that used in past case–control eBL studies is desirable to better characterize malaria serological patterns. Furthermore, previous studies on eBL only looked at IgG responses to both infections, but no measurement of IgM, as marker of primoinfection/current infection, was taken into account.

Here, we determined IgG and IgM serological signatures using a new multiplex panel combining 4 EBV and 15 *P. falciparum* antigens by qSAT in HIV-seronegative children from a cancer case–control study conducted in Malawi (11) to generate new hypotheses and get further insights into the interplay between EBV and *P. falciparum* infections in the development of eBL.

## Materials and Methods

### Study Participants

**Figure 1:**
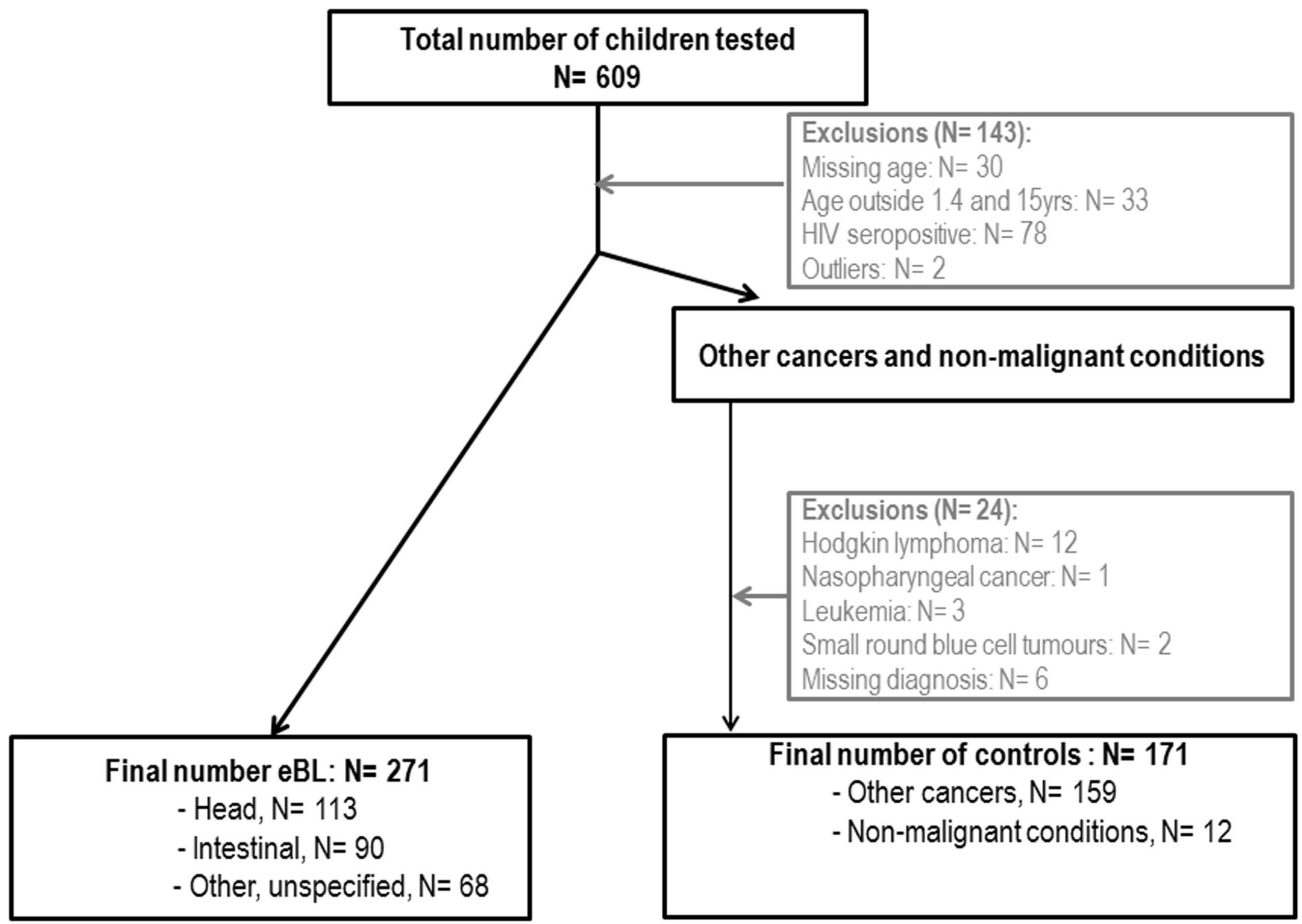
Flowchart of endemic Burkitt lymphoma (eBL) cases and controls included in the study.

Anonymized serum samples from 609 Malawian children were tested. Samples had been collected as part of a childhood malignancy and blood-borne virus study conducted at the Queen Elizabeth Hospital in Blantyre, Malawi, between 2005 and 2006 (11). Briefly, recruitment was performed in the pediatric oncology ward. The mother or guardian of each child was invited to participate in the study; written informed consent was provided, and standardized questionnaires administered. eBL was clinically diagnosed and in 75% of cases was confirmed by histology. HIV seropositives and infants <1 year who might have maternal IgGs were excluded from the current analysis. Overall, 271 eBL cases and 171 controls between 1.4 and 15 years old were analyzed (Figure 1). Controls included 159 children diagnosed with Wilms tumor (*n* = 60), retinoblastoma (*n* = 11), rhabdomyosarcoma (*n* = 28), Kaposi sarcoma (*n* = 7), neuroblastoma (*n* = 13), hepatocellular carcinoma (*n* = 13), malignant teratoma (*n* = 3), bone tumor (*n* = 5), brain tumor (*n* = 3), ovarian tumor (*n* = 4), yolk sac tumor (*n* = 3), skin carcinoma (*n* = 2), salivary glands tumor (*n* = 1), metastasis (*n* = 1), and other soft tissue tumors (*n* = 5). Additional 12 controls were children admitted to the oncology ward with a provisional diagnosis of cancer, who were subsequently found to have non-malignant conditions. All children diagnosed with hematological cancers (including leukemia, non-Burkitt lymphoma, and small round blue cell tumors), Hodgkin lymphoma or nasopharyngeal cancer were excluded from the control group because of possible diagnostic overlap and/or previously known strong associations with EBV. Among eBL cases, 113 children presented with head tumors and 90 with abdominal tumors.

### Ethics Statement

Ethical approval for the study was obtained from the Oxford Tropical Research Ethics Committee and the Malawian College of Medicine Research and Ethics Committee. The parent or guardian of each child provided written informed consent for their child to be included in the study in accordance with the Declaration of Helsinki.

### Laboratory Procedures

#### EBV GST-Fusion Proteins

Viral capsid antigen p18 subunit (VCA-p18), early antigen-diffuse component (EA-D), EBV nuclear antigen 1 (EBNA-1), and Z Epstein–Barr replication activator protein (ZEBRA) (12, 14) were expressed with pGEX vectors (Amersham) in *Escherichia coli* as double fusion proteins with N-terminal GST and a C-terminal peptide (tag). The proteins were affinity purified by incubation of glutathione displaying beads in bacterial lysate (15).

#### *P. falciparum* Recombinant Proteins

The antigens selected were based on their immunogenicity, their role in malaria pathogenesis and their presence in different parasite stages, some of them being vaccine candidates. Preerythrocytic antigens included the cell-traversal protein for ookinetes and sporozoites (CelTOS) (16), LSA-1 (17), sporozoite surface protein 2 (SSP2) also known as thrombospondin-related adhesion protein (18), and CSP (19), supplied by Protein Potential (USA). Blood-stage antigens included AMA-1 3D7 (20), receptor-binding region F2 of the 175 kDa erythrocyte binding antigen (F2) (EBA-175) (21), duffy binding-like (DBL)-α domain of *Pf*EMP-1 (22), DBL3X domain of VAR2CSA *Pf*EMP-1 (23), merozoite surface protein 3 (MSP-3) 3D7 (24), *Plasmodium* thrombospondin-related apical merozoite protein (PTRAMP) (25), reticulocyte binding-like homolog proteins 2 (Rh2) (26) and 5 (Rh5) (27), and cysteine-rich protective antigen 2 (CyRPA2) (28), produced at ICGEB. AMA-1 FVO (29) and MSP-1_42_ 3D7 and FVO (30) were produced at WRAIR.

#### Measurement of Antibodies to EBV and *P. falciparum* Proteins by qSAT

A multiplex panel was established to quantify IgGs and IgMs against *P. falciparum* and EBV antigens using xMAP™ technology (Luminex Corp., Austin, TX, USA) (31). Distribution of eBL cases and controls was balanced through plates. Briefly, 1,000 microspheres were used per analyte and sample. EBV proteins were affinity purified on GSH-bearing beads, thus for EBV antigens, a fusion protein consisting of GST and tag without intervening viral antigen was coupled to beads to be used for background determination. *P. falciparum* proteins were covalently coupled directly to the beads and beads were blocked with BSA; thus for *P. falciparum* antigens, BSA coupled to the beads was used for background determination. Several plasma pools were used as positive controls in each plate: (i) a *P. falciparum* hyperimmune Mozambican adult volunteers’ pool at 1/500 for IgG and 1/200 for IgM; (ii) an EBV immune Greek children pool (32) at 1/500 for IgG; and (iii) an EBV immune Malawi children pool (of samples with high IgM levels previously selected from the samples to be analyzed) at 1/200 for IgM. Several individual samples from malaria naïve European adults and non-EBV-exposed Greek children (32) were used as negative controls at 1/500 for IgG and 1/200 for IgM. Study samples were tested in duplicates at 1/500 and 1/20,000 for IgG and 1/200 and 1/20,000 for IgM. Two wells per plate were assayed without plasma added as blank controls. To block antibodies directed against residual bacterial proteins and GST present on the beads loaded with EBV proteins, plasmas were preincubated at a 1:20 dilution on a shaker at room temperature (RT) for 1 h in a serum preincubation buffer based on PBS, 1% BSA, 0.05% azide pH 7.4 containing 2 g/L lysate from bacteria expressing GST alone (15). After sample preincubation, beads were incubated with plasma samples for 1 h. The plate was washed by pelleting microspheres using a magnet and resuspended with 0.05% Tween 20-PBS. One hundred microliters of biotinylated antihuman IgG or IgM (Sigma, Tres Cantos, Spain) at 1/2,500 and 1/1,000, respectively, in PBS 1% BSA 0.05% azide pH 7.4 (assay buffer), were applied and incubated for 45 min. The plate was washed as before, and 100 µL of streptavidin-conjugated R-phycoerythrin (Invitrogen, Carlsbad, CA, USA) at 1/1,000 in assay buffer was added and incubated for 30 min. All incubations were done at RT with plate agitation (600 rpm) and protection from light. The plate was read using *Luminex 200™* analyzer and *Xponent* software version 3.1, and at least 50 microspheres per analyte were acquired per sample. Median fluorescence intensity (MFI) with blank fluorescence subtracted was exported, and arbitrary units (AU) concentration for each antigen was calculated by extrapolation from an IgG or IgM standard curve prepared by coupling beads to commercially available purified antihuman IgG and antihuman IgM specific to the F(ab′)_2_ region, and performing the assay using purified human IgG and human IgM as primary antibody.

#### Preprocessing of qSAT Data

Standard curves were fitted using four or five-parameter logistic equations depending on the best fit. The sample dilution closer to the EC_50_ of the curve was used to calculate the concentration (AU) as: EC_50_ (((*E*_min_ − *E*_max_)/(MFI − *E*_max_))^((1/Asym) − 1)^(1/Hill)), where EC_50_ is the half maximal effective concentration, *E*_min_ is the minimum response, *E*_max_ is the maximum response, Asym is the asymmetry factor, and Hill is the slope. Final concentrations were corrected by the dilution factor.

Seropositivity per antigen was defined as AU above the mean + 3 SDs of negative controls [non-malaria-exposed adults for *P. falciparum* and non-EBV-exposed children for EBV (32)]. EBV seropositivity was defined as positive IgGs against VCA-p18, EBNA-1 and EA-D, and positive IgM against VCA-p18 (33). *P. falciparum* seropositivity was defined as presence of antibodies to any of the parasite antigens.

### Statistical Analysis

Raw Luminex data (AU) were log transformed and standardized to normalize and bring all of the variables into proportion with one another (with mean 0 and SD 1). All analyses were done primarily using continuous data to gain power and avoid loss of information (34). Correlations were examined by Pearson matrices. Associations between serological responses to each antigen and demographic factors were examined using linear regression models adjusted for age and sex, as appropriate. Principal component analysis (PCA) was performed with an orthogonal rotation on EBV and *P. falciparum*, separately, to obtain the structure of the data for each infection (Table S1 in Supplementary Material). Two and five components were identified for EBV and *P. falciparum*, respectively. Based on PCA loading results, the two derived EBV components were labeled as “IgM EBV pattern” and “IgG EBV pattern.” Classical and multinomial logistic regression models were used to study the associations of antibodies, extracted principal components, and multiple seropositivity, with eBL. All models were adjusted for age (three categories: ≥1.41 < 3.75, ≥3.75 < 7.64, and ≥7.64 years) and sex. Further adjustment was performed including “IgG EBV pattern” (as continuous).

Elastic-net regression models were used to build an antibody response signature associated with eBL accounting for the high correlation and high dimensional scenario. Tuning elastic-net parameters (α and λ) were optimized using a 10-fold cross-validation minimizing the deviance. The stability of the selected variables was assessed using bootstrap with replacement (number of iterations *N* = 500) with the pair of parameters (α, λ) optimized again at each bootstrap iteration. Variables were considered as highly important if their inclusion frequency was ≥70%.

Both multiplicative and additive interactions (relative excess risk due to interaction RERI and synergy index S) were examined. As this is a hypothesis-generating study, we did not adjust for multiple comparisons, and results were interpreted for internal coherence and biological plausibility. Analyses were performed using STATA13 (35) and R (36) using the glmnet (37), c060 (38), and NMF packages (39).

## Results

### Demographic Characteristics of Study Participants

Table 1 shows demographic characteristics of participants. Controls were younger than cases [mean age (±SD): 6.3 (±3.7) and 7.8 (±2.9) years, respectively], with a proportion of children age of <4 years being much higher. Cases were more likely to be in the lower body mass index (BMI) tertile compared with the controls (51 vs 34%, respectively). The majority of cases and controls came from the South of Malawi (96 vs 87%, respectively). No differences were observed for other co-variables assessed.

**Table 1:** Basic demographic characteristics of study participants.

	Controls *N* (%)	Cases *N* (%)	*P*-het
	*N* = 171	*N* = 271	
**Age (years)**			
≥1.41 < 3.75	57 (33%)	16 (6%)	
≥3.75 < 7.64	57 (34%)	124 (46%)	
≥7.64	57 (33%)	131 (48%)	**<0.0001**
Mean age (SD)	6.3 (3.7)	7.8 (2.9)	**<0.0001**
Range	1.5–14.9	1.4–14.6	
**Sex**			
Female	75 (44%)	110 (41%)	
Male	99 (56%)	161 (59%)	0.50
**Body mass index (kg/m^2^)**			
First tertile (<14.18)	54 (34%)	129 (51%)	
Second tertile (≥14.18 ≤ 15.97)	53 (33%)	87 (34%)	
Third tertile (≥15.97)	53 (33%)	37 (15%)	**<0.0001**
**Country of residence**			
Malawi	162 (95%)	254 (94%)	
Mozambique	9 (5%)	16 (6%)	0.77
**Region**			
Central	17 (10%)	9 (4%)	
Northern	4 (2%)	1 (0%)	
Southern	142 (87%)	244 (96%)	**0.003**
**Guardian at interview**			
Mother	135 (79%)	204 (76%)	
Others	36 (21%)	66 (24%)	0.41

*Numbers may not add to the total because of missing values*.

*N, number; P-het: P-heterogeneity*.

*The numbers in bold correspond to the associations that are statistically significant at the 5% level*.

### Serological Characteristics of Study Participants

Overall, 88% of children were seropositive for EBV: 77% of controls and 96% of cases. Proportions by age group (≥1.41 < 3.75, ≥3.75 < 7.64, and ≥7.64 years) were 79, 75, and 77% among controls, and 88, 96, and 95% among cases. There were no differences in EBV seropositivity by sex (86% males vs 90% females). For *P. falciparum*, 99% children were IgG seropositive (99% cases and 98% controls) and 84% IgM seropositive (85% cases and 82% controls). Among controls, for most EBV and *P. falciparum* antigens, increasing age was associated with increasing antibody levels except for IgG VCA-p18 (Table S2 in Supplementary Material). Males tended to have lower IgM responses to several malaria antigens. No pattern was observed in the serological response by geographical region or by children’s BMI.

### “IgG EBV Pattern” and IgG against *P. falciparum* MSP-3 Antigen Are Associated with eBL

**Figure 2:**
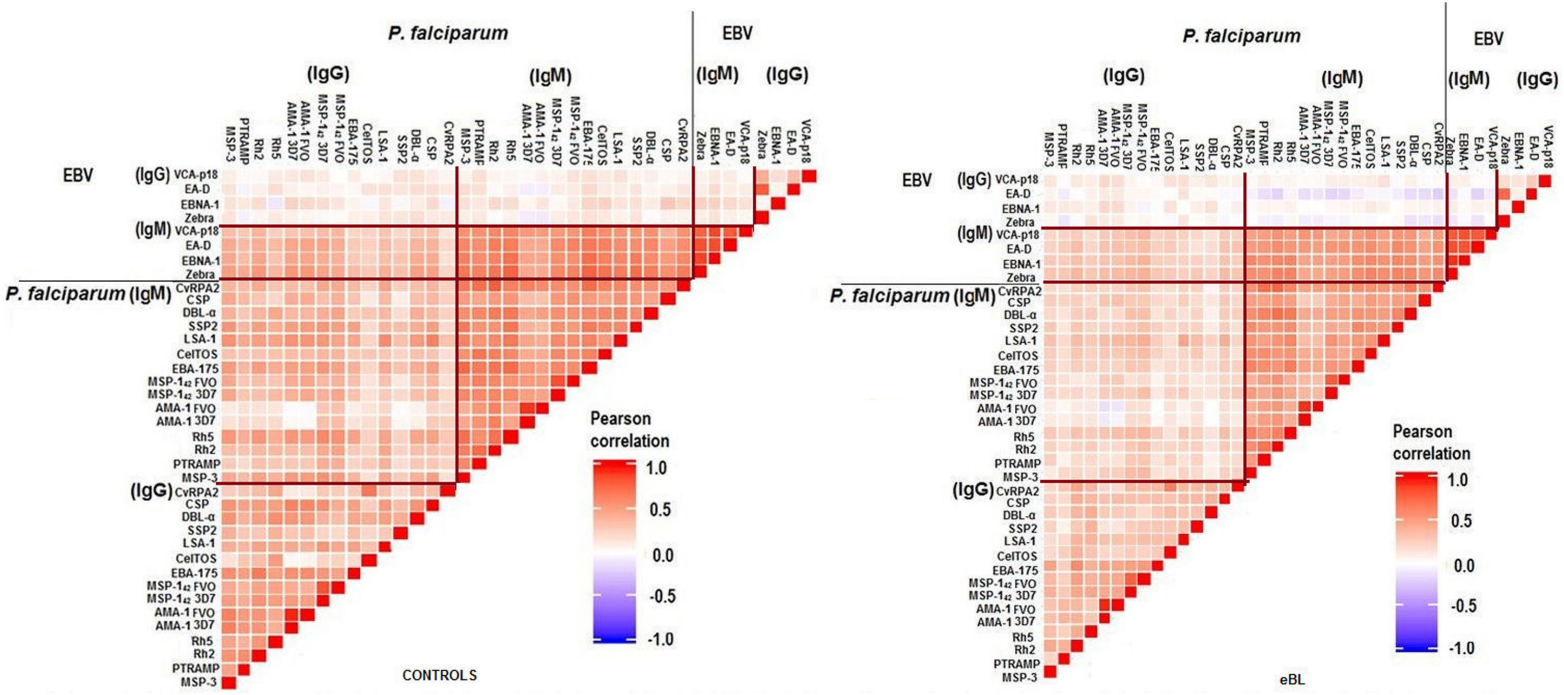
Pairwise correlations between logged antibody levels of both pathogens.

**Figure 3:**
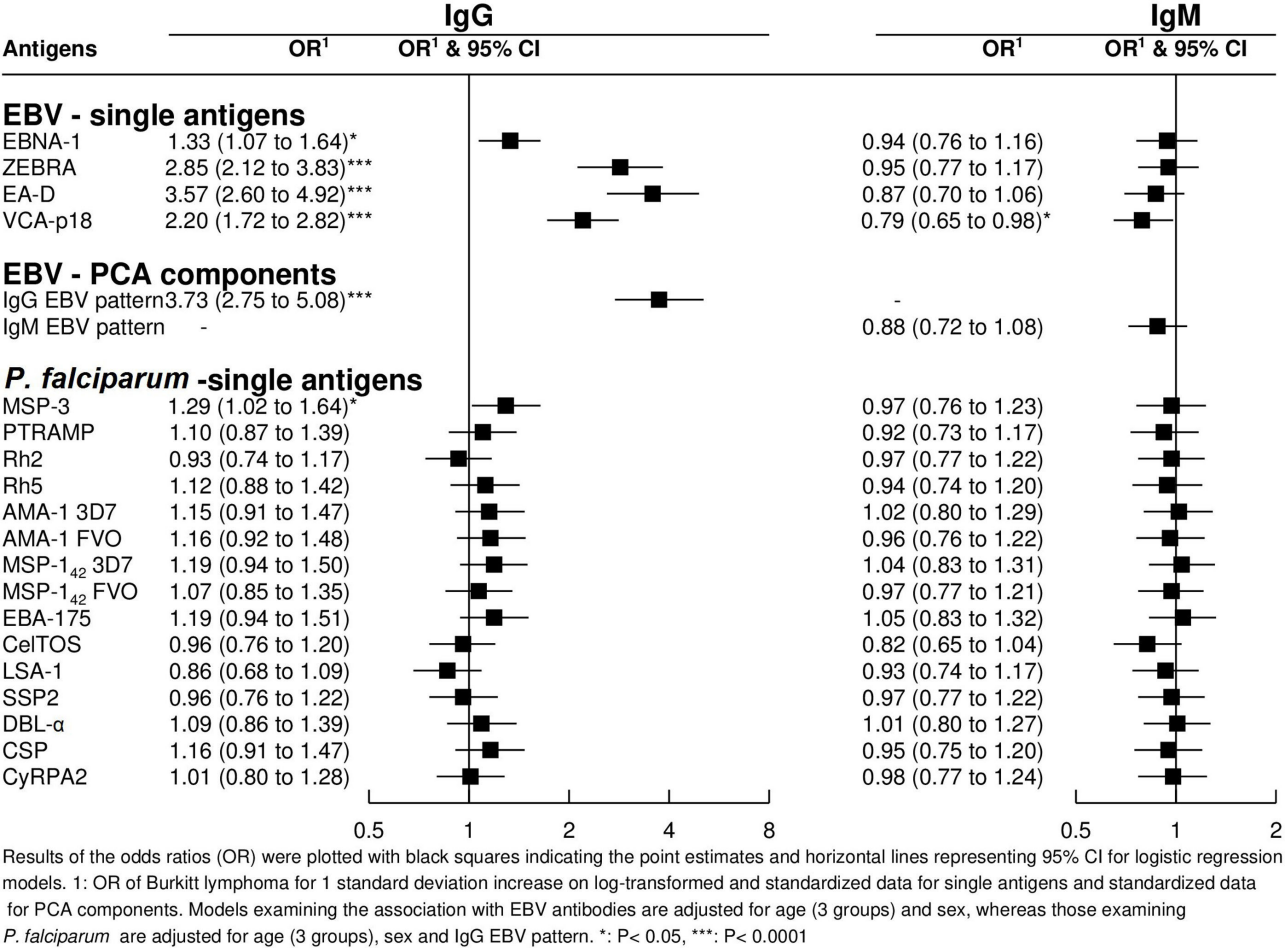
Odds ratios (ORs) for endemic Burkitt lymphoma in relation to IgG and IgM levels against single Epstein–Barr virus (EBV) and *Plasmodium falciparum* antigens and against EBV PCA components.

Strong correlations were observed between and within antibodies for both pathogens, especially between IgMs. Overall correlations tended to be stronger in controls than in cases (Figure 2). As expected, IgG to specific EBV antigens and the PCA component “IgG EBV pattern” were strongly positively associated with eBL {adjusted odds ratio (OR) [95% confidence interval (CI) = 3.73 (2.75–5.08)]; Figure 3}. On the contrary, IgMs to EBV were not associated with eBL, except for IgMs against VCA-p18 that were negatively associated with eBL after adjusting for age and sex.

In relation to *P. falciparum* antigens, after controlling for age, sex, and “IgG EBV pattern,” positive associations were observed for IgG against MSP-3 (Figure 3). In contrast to EBV results, none of the five PCA components derived from *P. falciparum* serology were associated with eBL after controlling for age, sex, and “IgG EBV pattern” (data not shown).

### IgGs against Multiple *P. falciparum* Erythrocytic Antigens Weakly Associated with eBL

Overall, joint presence of antibodies to multiple *P. falciparum* antigens was not associated with eBL after adjustment for age, sex, and “IgG EBV pattern” (Table 2). When antibodies were further grouped following parasite stages, a positive association was observed for the joint presence of IgGs to erythrocytic antigens [OR (95% CI) for seropositivity to third tertiles = 1.04 (1.00–1.09)] but not for IgGs to preerythrocytic antigens. No associations were observed for joint presence of multiple IgMs (data not shown).

**Table 2:** ORs of endemic Burkitt lymphoma for the burden of IgGs to *Plasmodium falciparum* antigens.

Risk factors	Controls *N* (%)	Cases *N* (%)	OR^a^ (95% CI)	*P*-trend	OR^b^ (95% CI)	*P*-trend
**All *P. falciparum* antigens**						
Lower^c^	58 (34)	42 (15)	Ref		Ref	
Medium	58 (34)	125 (46)	2.44 (1.42–4.21)		1.72 (0.94–3.17)	
Higher	55 (32)	104 (38)	1.89 (1.08–3.29)	0.06	1.39 (0.75–2.58)	0.40
1 U increase			**1.04 (1.01–1.07)**	**0.005**	1.02 (0.99–1.05)	0.18
**Preerythrocytic antigens**						
Lower^c^	76 (44)	99 (37)	Ref		Ref	
Medium	41 (24)	76 (28)	1.26 (0.75–2.12)		1.00 (0.56–1.81)	
Higher	54 (32)	96 (35)	1.08 (0.67–1.76)	0.72	0.81 (0.47–1.41)	0.47
1 U increase			1.03 (0.93–1.13)	0.58	0.97 (0.87–1.08)	0.56
**Erythrocytic antigens**						
Lower^c^	60 (35)	36 (13)	**Ref**		Ref	
Medium	59 (35)	121 (45)	**2.71 (1.55–4.74)**		1.87 (1.00–3.48)	
Higher	52 (30)	114 (42)	**2.60 (1.48–4.60)**	**0.003**	1.85 (0.98–3.49)	0.08
1 U increase			**1.07 (1.03–1.11)**	**0.001**	**1.04 (1.00–1.09)**	**0.05**

*Ref, reference; OR, odds ratio; CI, confidence interval*.

*^a^Adjusted for age (three categories) and sex*.

^b^Adjusted for age, sex, and the principal component analysis component “IgG Epstein–Barr virus pattern.”

*^c^The burden was calculated creating a score by adding tertiles values of the log-transformed data (coded as 1, 2, and 3) for each antigen and dividing the score into three groups based on the control distribution. Preerythrocytic antigens: CelTOS, SSP2, CSP and LSA-1*.

*The numbers in bold correspond to the associations that are statistically significant at the 5% level*.

### Antibody Response Signature for eBL

**Figure 4:**
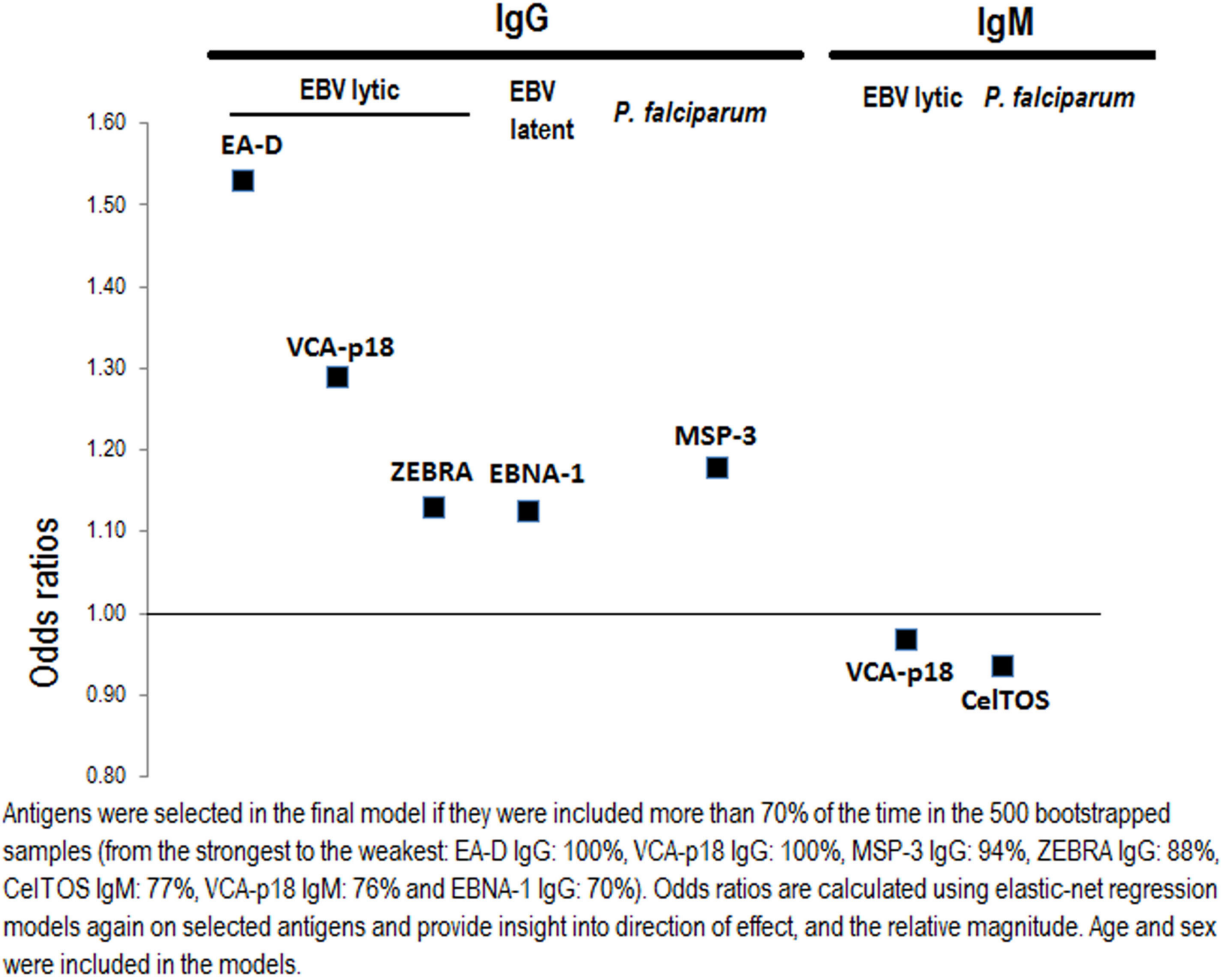
Selecting important Epstein–Barr virus (EBV) and *Plasmodium falciparum* antigen-specific antibodies for endemic Burkitt lymphoma using elastic-net penalized regression models.

The antibody response signature for eBL included antibodies to 7 antigens out of the 38 examined (Figure 4). Highest eBL positive ORs were observed for increasing IgGs against the three EBV lytic antigens EA-D, and VCA-p18 (OR = 1.53 and 1.29, respectively), and to a lesser extent for increasing IgG to the latent antigen EBNA-1 and the lytic antigen ZEBRA (OR = 1.13 for both antigens). Regarding the IgM EBV repertoire, OR of eBL decreased with increasing IgM to VCA-p18 (OR = 0.97). In relation to Igs to *P. falciparum*, children with eBL were more likely to have higher IgG against MSP-3 (OR = 1.18) and lower IgM against CelTOS (OR = 0.94).

### Additive Interaction between IgGs against EBV and MSP-3

**Figure 5:**
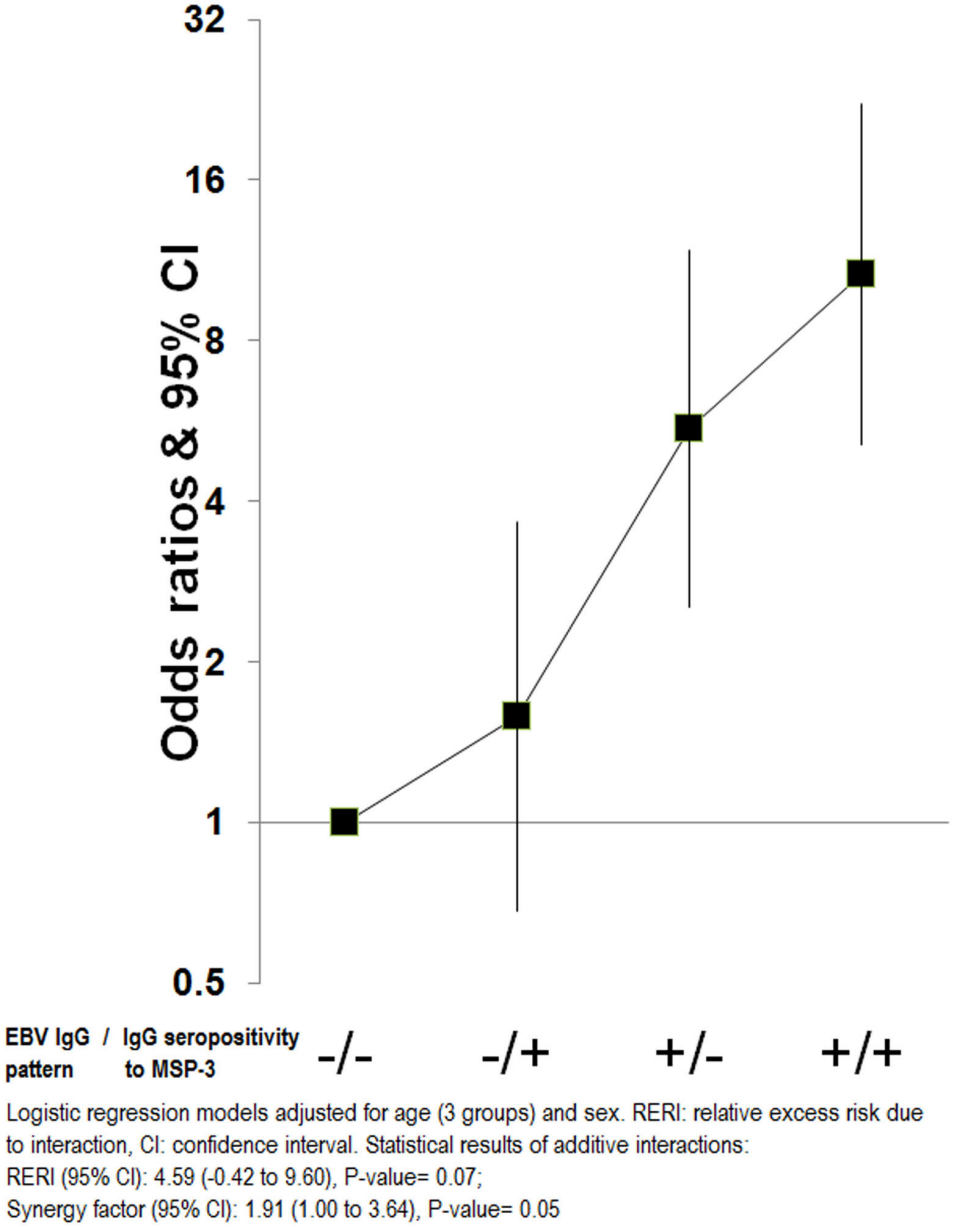
Additive interactions for endemic Burkitt lymphoma between “Epstein–Barr virus (EBV) IgG pattern” and IgG to merozoite surface protein 3 antigen.

An additive effect of the “IgG EBV pattern” and IgG seropositivity to MSP-3 was observed that significantly increased the OR of eBL beyond the associated with each factor independently [OR (95% CI) for high “IgG EBV pattern” and IgG seropositivity to MSP-3 compared with low/seronegativity = 10.6 (5.1–22.2)]. However, this interaction reached borderline statistical significance (*P* = 0.05) (Figure 5). No other interactions were observed.

### Association between Antibody Responses and Tumor Location

**Figure 6:**
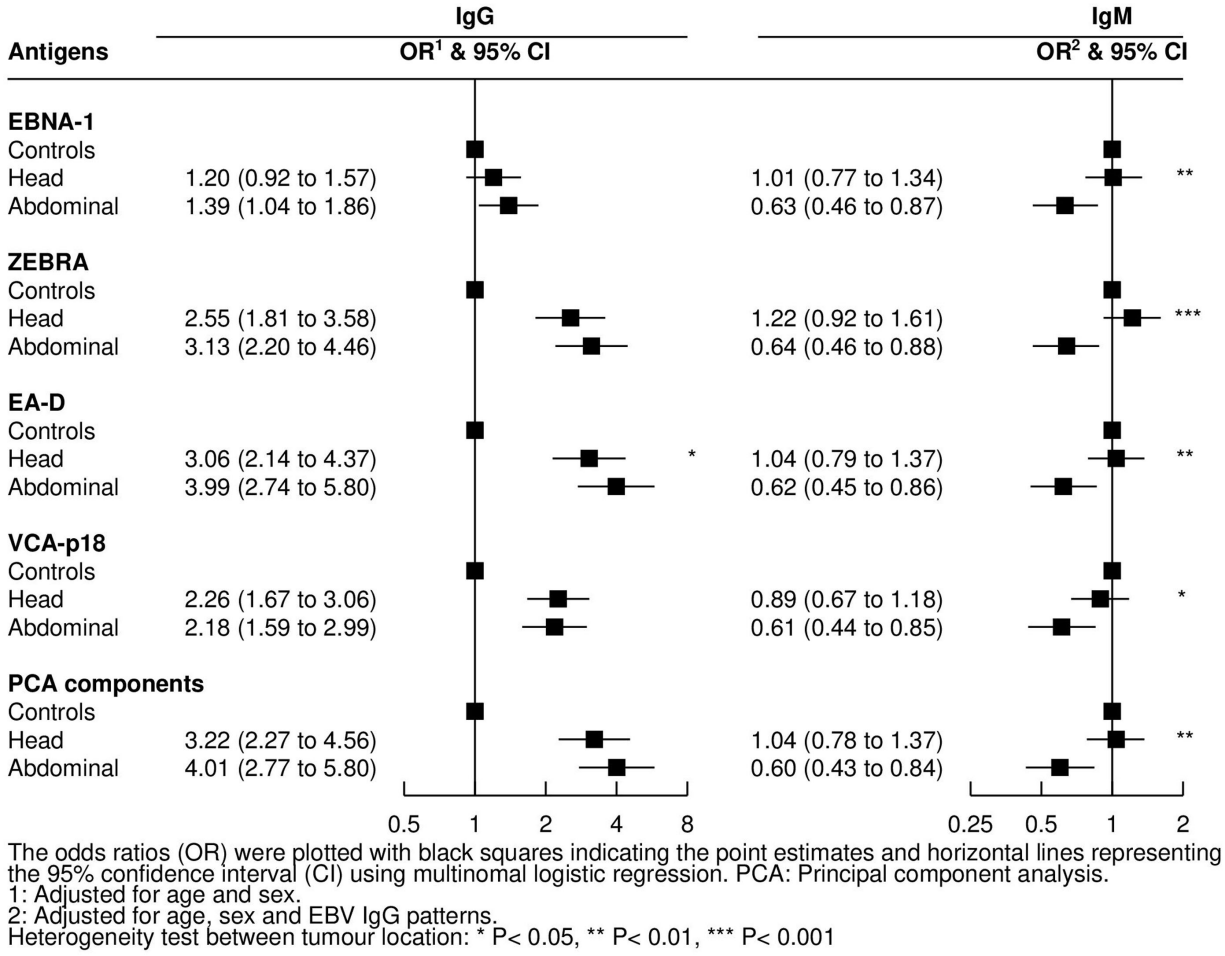
Adjusted odds ratios (ORs) and 95% confidence intervals (CIs) for endemic Burkitt lymphoma in relation to IgG and IgM levels against Epstein–Barr virus (EBV) antigens by tumor location.

Cases with abdominal tumors were older than cases with jaw tumors [mean (SD) age: 8.9 (0.3) vs 6.9 (0.2) years, respectively; *P* < 0.001] and more likely to be female (47 vs 32%, *P* = 0.03). The association between tumor location and the assessed antibodies as well as PCA compounds was examined for both infections (Figure 6). Compared with controls, OR for abdominal tumors decreased with increasing IgM against EBV, whereas no difference was observed in children with head tumors (all *P*-values for heterogeneity between tumor location <0.05). Regarding IgGs to EBV antigens, only IgG to EA-D was positively associated with higher OR for abdominal tumors compared with head tumors (*P* = 0.05). No associations were observed for antibodies to *P. falciparum* and tumor location (data not shown).

## Discussion

This study aimed to give further insight into the combined effect of EBV and *P. falciparum* in the development of eBL by analyzing IgG and IgM patterns to 4 EBV and 15 *P. falciparum* antigens (10 never used before, including 3 preerythrocytic) in Malawian children from a cancer case–control study (11). As expected, our data support a strong role of EBV in eBL, and a weak effect of malaria, but suggest that both infections might act jointly on eBL, as shown by the additive interaction between elevated IgGs against EBV and MSP-3. In agreement with this observation, two recent studies have reported that the key determinants of EBV primary infection kinetics in children are the degree of malaria exposure and maternal antibody decay (9, 40).

The study population showed a high EBV seroconversion at a young age (>3/4 of the controls age ≤3.8 years), contrasting with recent estimates from Crete where only 50% of children at age 4 years had seroconverted (32). As expected, elevated EBV IgG levels were strongly positively associated with eBL (10–12, 41), whereas high anti-VCA-p18 IgM responses were negatively associated with eBL. In particular, IgG levels to the three EBV lytic antigens (EA-D, marker of active infection and viral reactivation, ZEBRA involved in the switch from latent to lytic stage, and VCA-p18) were higher compared with IgG against EBNA-1 (latency marker). High IgM levels against VCA-p18 usually indicate recent primary EBV infection, although it may persist for months or might reappear in EBV reactivation. The negative association of high IgM anti-VCA-p18 levels with eBL suggests that newly EBV-infected children might be at lesser risk of eBL compared with children exposed for a longer time, or might indicate that in a malaria endemic region the long-term persistence of anti-VCA IgM responses may be associated with a better control of EBV infection (40).

In contrast to the strong EBV association, we only observed weak associations for few of the 15 antibodies measured against *P. falciparum* antigens, which is difficult to interpret as serological understanding of *P. falciparum* exposure and acquired immunity are not yet fully elucidated (42). However, the observed differences are probably related to the diverse immunogenicities of these antigens. The strongest and most consistent association was found for IgG against MSP-3, a highly immunogenic antigen involved in merozoite binding to human erythrocytes (43). Anti-MSP-3 IgG responses have been associated with protection against malaria in some sero-epidemiological studies (44, 45), but in others just showed to be exposure markers (46, 47). Higher burden of IgGs against multiple merozoite proteins was observed in eBL patients compared with controls suggesting that a persistent/chronic malaria exposure might enhance EBV infection as well as reactivation. To deal with the issue of assessing associations for correlated EBV and *P. falciparum* antigen-specific antibodies, we used elastic-net regression models to select a subset of the most important antibodies for eBL, mutually adjusted for each other. While getting inference testing on the selected antigens for elastic-net is still an open statistical question, the results allow us to generate some new hypotheses. The higher IgM responses to the liver-stage antigen CelTOS were inversely associated with eBL, suggesting that newly malaria-infected children might be at lesser risk of eBL compared with children exposed for a longer time. Alternatively, the long-term persistence of anti-CelTOS IgM responses may be associated with a better control of malaria infection. CelTOS-specific cellular immunity has been detected among protected subjects in a controlled human malaria infection study (48), and antibodies to CelTOS have been shown to correlate with naturally acquired clinical immunity (49). Also, a recent study reports that anti-CelTOS responses prevent hepatocyte infection by sporozoites (50).

As secondary analysis, we observed that lower ”EBV IgM pattern” and higher IgG responses to EA-D occurred in children with abdominal tumor presentation compared with head location. This differs to results from a previous study (*N* = 14 jaw and 16 abdominal tumors) where higher anti-ZEBRA but not anti-EA-D IgG levels were associated with abdominal tumors (12). Here, IgG levels to EA-D highly correlated with ZEBRA IgG, particularly among cases, and consequently both IgG responses to ZEBRA and EA-D were elevated in children with abdominal vs head tumors, but results were only statistically significant for EA-D. We also observed that children with abdominal tumors were more likely to have lower EBV IgMs, suggesting that abdominal compared with jaw tumors may arise more frequently in long-standing EBV infections. In turn, this longer EBV exposure could explain the observed age difference between distinct clinical presentations, with abdominal eBL occurring more often among older children.

Our study has some limitations. First, results are based on case–control data, so temporality of infections and causality of associations cannot be determined. Thus, the observed associations with the two infections could be due to reverse causality since changes in antibody levels could be consequence of eBL disease. The use of hospital oncologic controls might be another limitation. Although we excluded all children diagnosed with hematological cancers, Hodgkin lymphoma or nasopharyngeal tumors, to avoid possible diagnostic overlap with eBL, and to exclude those with known associations with EBV, we cannot discard that some of these other conditions might be affecting the antibody responses to EBV and *P. falciparum* antigens. Misclassification in cases and tumor location might have occurred, as 25% of cases and controls did not have a histological verification of diagnosis; children might have had metastasis at hospital arrival, making the classification difficult. In addition, the understanding of the sero-epidemiology of *P. falciparum* is at an early stage, making the interpretation of the findings challenging. It would have been informative to examine the serological responses in relation to EBV and parasite densities, but this information was not available. However, the elevated levels of IgG to EA-D reflect an increased production of EBV DNA and hence might be a surrogate of EBV-viral load. Finally, multiple associations were evaluated and chance in some of them cannot be excluded, thus the weak associations observed for some *P. falciparum* antigens with eBL and the additive interaction of MSP-3 with EBV must be taken with caution. Nevertheless, the confirmation of previous findings and the internal consistency of results make us confident that the findings are robust.

In conclusion, we found a strong association between EBV and eBL, and identified new associations between specific antibodies to *P. falciparum*, EBV, and eBL. Multiple *P. falciparum* antibody responses and differential effects by liver- vs blood-stage infections might reflect a complex mechanism whereby the parasite modulates the association between EBV and eBL. Longitudinal studies with more immunological and molecular data on EBV and *P. falciparum* infections are needed to better understand the combined mechanism of the two infections in eBL etiology. Future studies on eBL should collect information on tumor location since the infections might act differently by tumor presentation. The development of vaccines against these two infections would be crucial to protect African children from eBL.

## Ethics Statement

Ethical approval for the study was obtained from the Oxford Tropical Research Ethics Committee and the Malawian College of Medicine Research and Ethics Committee. The parent or guardian of each child provided written informed consent for their child to be included in the study in accordance with the Declaration of Helsinki.

## Authors Contributions

Substantial contributions to the conception or design of the work: RA, DC, CO-G, JC, SS, TW, MK, RN, and CD. Acquisition of samples/data: NM, RN, EA, SD, DG, CC, VC, AM, and TW. Analysis of samples: RA, MV, JC, and TW. Analysis of data: DC. Interpretation of data: RA, DC, CO-G, SS, TW, MK, RN, and CD. Draft of the manuscript: RA and DC. Critical revision of the draft for important intellectual content, final approval of the version to be published, and agreement to be accountable for all aspects of the work in ensuring that questions related to the accuracy or integrity of any part of the work are appropriately investigated and resolved: all the authors.

## Conflict of Interest Statement

The authors declare that the research was conducted in the absence of any commercial or financial relationships that could be construed as a potential conflict of interest. The reviewer KF and handling editor declared their shared affiliation.
