# Supplementary material for: Assessment of the Combined Effect of Epstein–Barr Virus and Plasmodium falciparum Infections on Endemic Burkitt Lymphoma Using a Multiplex Serological Approach
Source: Front Immunol. 2017 Oct 26;8:1284. doi: 10.3389/fimmu.2017.01284 (PMC5662586; doi:10.3389/fimmu.2017.01284)
Supplement: Supplementary file 1 [file Data_Sheet_1.DOCX]

**Supplementary material**

**TABLES**

**T 1**. Factor loadings of 30% or greater using principal component analysis on antibody levels against EBV and *P. falciparum* antigens.

**T 2**. Factors associated with antibody levels to EBV and *P. falciparum* antigens among controls (N= 171).

**Table 1.** Factor loadings of 30% or greater using principal component analysis on antibody levels against EBV and *P. falciparum* antigens


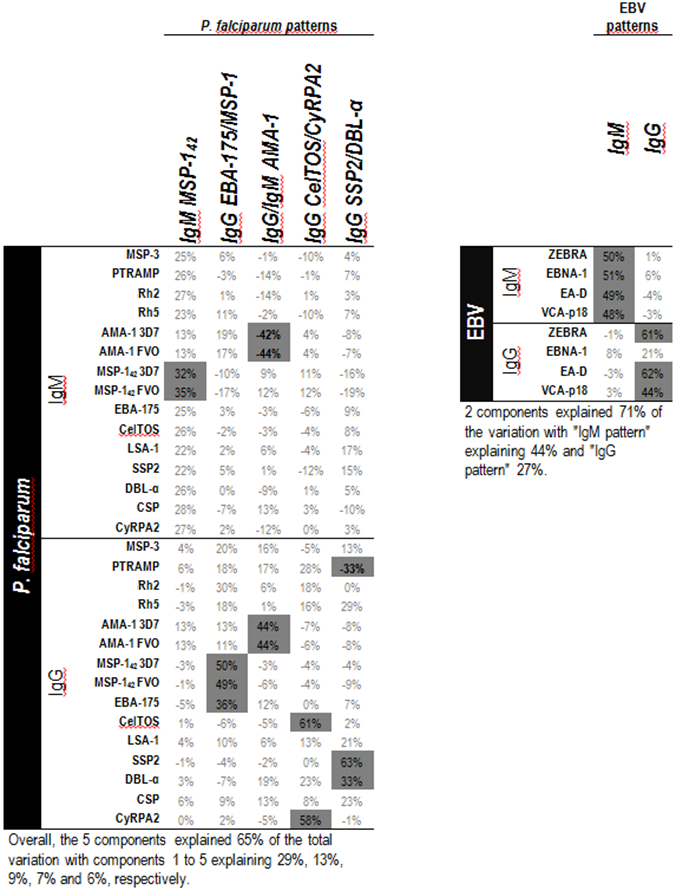


**Table 2.** Factors associated with antibody levels to EBV and *P. falciparum* antigens among controls (N= 171)

|  |  |  |  | | | |
| --- | --- | --- | --- | --- | --- | --- |
|  |  |  | **Age** | **Sex** | **BMI** | **Region** |
| **EBV** | **IgG** | **EBNA-1 (tr)** |  |  |  |  |
|  |  | **VCAp18** |  |  |  |  |
|  |  | **ZEBRA** | **↗*** |  |  |  |
|  |  | **Ea(D)** | **↗*** |  |  |  |
|  | **IgM** | **EBNA-1 (tr)** | **↗*** |  |  |  |
|  |  | **VCAp18** |  |  |  |  |
|  |  | **ZEBRA** | **↗*** | **↘*male** |  |  |
|  |  | **Ea(D)** | **↗*** |  |  |  |
|  |  |  |  |  |  |  |
| ***P. falciparum*** | **IgG** | **MSP-3** | **↗*** |  |  |  |
|  |  | **PTRAMP** |  |  |  |  |
|  |  | **Rh2** | **↗*** |  |  |  |
|  |  | **Rh5** | **↗*** |  |  |  |
|  |  | **AMA-1 3D7** | **↗*** |  | **↘*** |  |
|  |  | **AMA-1 FVO** | **↗*** |  | **↘*** |  |
|  |  | **MSP-1 42 3D7** | **↗*** |  |  |  |
|  |  | **MSP-1 42 FVO** | **↗*** |  |  |  |
|  |  | **EBA-175** | **↗*** |  |  |  |
|  |  | **CelTOS** |  |  |  |  |
|  |  | **LSA-1** | **↗*** |  |  |  |
|  |  | **SSP2** | **↗*** |  |  |  |
|  |  | **DBL-α** | **↗*** |  |  |  |
|  |  | **CSP** | **↗*** |  |  |  |
|  |  | **CyRPA2** |  |  |  |  |
|  | **IgM** | **MSP-3** | **↗*** | **↘*male** |  |  |
|  |  | **PTRAMP** |  | **↘*male** |  |  |
|  |  | **Rh2** |  | **↘*male** |  |  |
|  |  | **Rh5** | **↗*** |  |  |  |
|  |  | **AMA-1 3D7** |  |  |  |  |
|  |  | **AMA-1 FVO** |  |  |  |  |
|  |  | **MSP-1 42 3D7** |  | **↘*male** |  |  |
|  |  | **MSP-1 42 FVO** |  | **↘*male** |  |  |
|  |  | **EBA-175** | **↗*** | **↘*male** |  |  |
|  |  | **CelTOS** |  | **↘*male** |  |  |
|  |  | **LSA-1** | **↗*** |  |  |  |
|  |  | **SSP2** | **↗*** |  |  |  |
|  |  | **DBL-α** |  |  |  |  |
|  |  | **CSP** |  |  |  |  |
|  |  | **CyRPA2** |  | **↘*male** |  |  |

*: P< 0.05. Unadjusted linear models were used.

EBV: Epstein-Barr virus, BMI: body mass index.
